# Generating age-specific mortality statistics from incomplete death registration data: two applications of the empirical completeness method

**DOI:** 10.1186/s12963-021-00262-3

**Published:** 2021-06-07

**Authors:** Tim Adair, Alan D Lopez

**Affiliations:** 1 Melbourne School of Population and Global Health, The University of Melbourne, Level 5, Building 379, 207 Bouverie Street, Carlton, Victoria 3010 Australia; 2 Institute for Health Metrics and Evaluation, University of Washington, 2301 5th Ave, Seattle, WA 98121 USA

**Keywords:** Mortality, Vital statistics, Civil registration, Data quality, Completeness, Capture-recapture

## Abstract

**Background:**

The study aims to assess two approaches that apply the empirical completeness method to generate age-specific mortality statistics from incomplete death registration systems.

**Methods:**

We use the empirical completeness method to calculate all-age death registration completeness, which is used with a model life table to generate mortality statistics and age-specific completeness using (1) the conventional method and (2) the equivalent deaths method. The results are compared with a capture-recapture (C-RC) study and three alternative mortality estimates for Brazilian states, and C-RC studies in Thailand, Oman and Vietnam, which independently estimate the level and age pattern of mortality or completeness.

**Results:**

The empirical completeness method produces similar estimates of all-age completeness of registration to the C-RC studies. Compared with C-RC studies, at 15-59 years, the conventional method’s estimates of mortality and completeness are more concordant, while at 60-84 years the equivalent death method’s estimates are closer. Estimates of life expectancy from the two approaches each have similar concordance with the C-RC studies. For male adult mortality in Brazilian states, there is relatively strong average correlation of this study's estimates with three alternative estimates.

**Conclusions:**

The two approaches produce mortality statistics from incomplete data that are mostly concordant with C-RC studies, and can be most usefully applied to subnational populations.

**Supplementary Information:**

The online version contains supplementary material available at 10.1186/s12963-021-00262-3.

## Introduction

Routine, timely and accurate indicators of the level and pattern of mortality in a population are best informed by a complete civil registration and vital statistics (CRVS) system. Summary all-cause and cause-specific mortality indicators produced by CRVS data track progress towards national and subnational goals; 16 Sustainable Development Goals (SDGs) and 24 corresponding indicators require data that are most effectively produced by an accurate and timely CRVS system [1]. These mortality statistics are reliant on registered deaths disaggregated by age and sex that enable, for example, age-standardisation of rates and production of life tables. Analysis of the risk of mortality by age groups and sex can also reveal much of policy relevance about a population’s health, even in the absence of reliable cause of death data.

However, CRVS systems are incomplete in many countries, which limits their utilisation as evidence for health policy [2]. Use of unadjusted incomplete death registration data to generate mortality statistics will result in an under-estimation of these key mortality indicators. Measurement of the completeness of death registration is therefore important to understand how well deaths are captured by the CRVS system in national and subnational populations and to generate adjusted mortality statistics. Completeness of registration can be estimated using a range of methods, each with strengths and limitations; these include death distribution methods (DDMs), the empirical completeness method, and capture-recapture (C-RC) of deaths (or direct methods) [3–7]. The empirical completeness method, which models observed versus expected completeness based on modelling the key drivers of mortality levels in a population, has advantages over DDMs, including the use of relatively limited data that are readily available at the national and subnational levels, timely estimates according to when the most recent CRVS data are available (compared with the most reliable DDMs that estimate completeness for the most recent intercensal period) and a lack of reliance on often unrealistic assumptions about population dynamics (e.g. closed migration) that can especially bias subnational estimates [7].

DDMs and the empirical completeness method estimate a constant level of completeness above 5 years of age (or all ages for the empirical completeness method), and in isolation do not reveal information on age patterns of mortality that are necessary to produce mortality statistics [5–10]. A conventional method to generating life tables from incomplete death registration data is to estimate completeness of registration, calculating the _*45*_*q*_*15*_ (probability of dying between age 15 and 60 years, or adult mortality) from death registration data which is then adjusted using this level of completeness, and inputting the adjusted _*45*_*q*_*15*_ and an estimate of _*5*_*q*_*0*_ (probability of dying from live birth to 5 years) into a model life table (MLT) [5]. MLTs represent the global range of levels and age-patterns of mortality rates, and are used to generate complete life tables from such data of suboptimal quality [11]. Another source of estimates of deaths, by age and sex, from which completeness can be calculated are the Global Burden of Disease (GBD) and United Nations World Population Prospects (UNWPP), which are produced for all countries [12, 13]. Both the GBD and UNWPP also estimate _5_*q*_0_ and _*45*_*q*_*15*_ from a range of available data using various methods (including completeness-adjusted registration data) and input these into MLTs. A potential drawback of this general approach to adjusting _*45*_*q*_*15*_ is that if the level of completeness at 5 years and above used to adjust deaths at ages 15-59 years is different from the true completeness for 15-59 years, then the adjusted _*45*_*q*_*15*_ and subsequent final life table will be inaccurate. A further limitation is that the MLT may not reflect the true age pattern of local mortality.

C-RC methods, which match individual death data in a death registration (or reporting) system to another data source, can however estimate completeness of death registration for specific age groups and use this to adjust registration data and produce complete life tables [3, 14]. Importantly, C-RC studies’ estimates of age-specific completeness of registration (and hence the true age pattern of mortality) do not rely on an assumed age pattern of mortality from a MLT. Reliable application of C-RC methods requires having two independent sources of data that can be successfully linked. However, many C-RC studies are time- and resource-intensive due to the data linkage process and some studies have required a new data collection independent from the registration system to be undertaken. Hence, C-RC studies of mortality registration data are relatively rare. Recent examples of such studies include those conducted in Brazil, Thailand, Vietnam, Brazil, Oman, Kiribati and Bohol, Philippines [14–20].

In summary, there is potential error in mortality statistics generated from incomplete death registration data. However, given the advantages of the empirical completeness method in estimating all-age completeness, it is worthwhile to assess its application to generate age-specific mortality statistics. This study examines two different methods that apply the empirical completeness method to generate mortality statistics and which make different assumptions about completeness at 5 years and above:

1. Conventional method: Adjusting _*45*_*q*_*15*_ using completeness at ages 5 years and above, and inputting the adjusted _*45*_*q*_*15*_ with _*5*_*q*_*0*_ into an MLT.
2. Equivalent deaths method: Integrating the empirical completeness method with an MLT to ensure that they each produce an equal estimated total number of deaths for each sex (i.e. not restricting the _*45*_*q*_*15*_ to be adjusted based on completeness at age 5 years and above).

The study compares mortality statistics and age-specific completeness according to these methods with C-RC studies and three alternative mortality estimates from Brazilian states, and C-RC studies from Thailand, Vietnam and Oman. C-RC methods in particular are a valuable comparator because they independently measure age patterns of mortality and completeness without reliance on MLTs. The study’s findings should improve the ability of analysts, particularly at the subnational level, to estimate timely and reliable mortality indicators in their populations using incomplete death registration data.

## Methods

### Empirical completeness method

The empirical completeness method estimates the completeness of death registration (or completeness of any routine death reporting system such as a Health and Demographic Surveillance System (HDSS) site) for all ages from the following models [7]:

$$ logit\ \left({C}_{jk1}^{All}\right)={\beta}_0+{RegCDR sq}_{jk}\times {\beta}_1+{RegCDR}_{jk}\times {\beta}_2+\%{65}_{jk}\times {\beta}_3+\mathit{\ln}{(5q0)}_{jk}\times {\beta}_4+{C}_{jk}^{<5}\times {\beta}_5+k\times {\beta}_6+{\gamma}_j $$ (Model 1)

$$ logit\ \left({C}_{jk2}^{All}\right)={\beta}_0+{RegCDR sq}_{jk}\times {\beta}_1+{RegCDR}_{jk}\times {\beta}_2+\%{65}_{jk}\times {\beta}_3+\mathit{\ln}{(5q0)}_{jk}\times {\beta}_4+k\times {\beta}_5+{\gamma}_j $$ (Model 2)

where ${C}_{jk1}^{All}$ is the completeness of registration at all ages in Model 1 and ${C}_{jk2}^{All}$ is the completeness of registration at all ages in Model 2, *logit*(${C}_{jk1}^{All}$) is $\mathit{\ln}\left(\frac{C_{jk1}^{All}}{1-{C}_{jk1}^{All}}\right)$ and *logit*(${C}_{jk2}^{All}$) is $\mathit{\ln}\left(\frac{C_{jk2}^{All}}{1-{C}_{jk2}^{All}}\right)$, *RegCDR*_*jk*_ is the registered crude death rate (CDR), *RegCDRsq*_*jk*_ is the square of *RegCDR*, %65_*jk*_ is the fraction of the population aged 65 years and over, *ln*(5*q*0)_*jk*_ is the natural log of the estimate of the true under-five mortality rate (this can be obtained nationally from the GBD or International Group for Mortality Estimation (IGME), or subnationally from Demographic and Health Surveys or censuses using summary or complete birth history data), ${C}_{jk}^{<5}$ is the completeness of the registered under-five mortality (estimated as the under-five mortality rate from registration data divided by the estimate of the true under-five mortality rate), *k* is calendar year (which captures changes in the relationship between independent and dependent variables in the model), *γ* is a country-level random effect , *j* is country and *β*_0_ to *β*_6_ are the coefficients. Predicted completeness is then estimated using the inverse logit, e.g. for Model 1: $\frac{e^{logit\left({C}_{jk1}^{All}\right)}}{e^{logit\left({C}_{jk1}^{All}\right)}+1}$ [7, 21].

### Estimating mortality indicators using the empirical completeness method and model life table with flexible standards (MLTFS)

#### MLTFS

This study uses the empirical completeness method with an MLT—the model life table with flexible standards (MLTFS)—to estimate complete life tables and hence mortality indicators. The MLTFS is a model life table system that generates a complete life table for a population using inputs of _5_*q*_0_, _*45*_*q*_*15*_ and a standard life table, and is the model life table system used by the GBD [22]. The MLTFS was developed based on over 7000 empirical life tables from the Human Mortality Database and vital registration data adjusted for completeness. The equation used (where there is no HIV) is as follows:

$$ logit\left({}_n{}{q}_x^c\right)= logit\left({}_n{}{q}_x^s\right)+{\beta}_x^1\left( logit\left({}_5{}{q}_0^c\right)- logit\left({}_5{}{q}_0^s\right)\right)+{\beta}_x^2\left( logit\left({}_{45}{}{q}_{15}^c\right)- logit\left({}_{45}{}{q}_{15}^s\right)\right) $$

where *c* denotes the country that the data are from (although it can denote any population the MLTFS is used for) and *s* is the standard life table [22]. Coefficients for the MLTFS are presented in Additional file 1: Table A.1. The standard life table in the GBD’s application of the MLTFS is either recent life tables of that country or, if these do not exist, a regional standard life table. Without a reliable standard such as the GBD country life table, the resultant mortality estimates might well be implausible [23, 24]. The MLTFS’s flexibility however allows for departures in _5_*q*_0_ and _45_*q*_15_ to estimate a complete life table which may differ significantly from the standard life table [22]. The resultant ${}_n{}{q}_x^c$from the MLTFS values can be converted to age-specific mortality rates (${}_n{}{m}_x^c$) using conventional life table calculations. At ages 85 years and over, the ${}_n{}{q}_x^c$values can be estimated using a Gompertz function [25].

For the application of the MLTFS with the empirical completeness method (detailed below), we recommend use of the national GBD life table as the standard life table, consistent with the GBD’s choice of standard life table and because it preserves the age pattern of mortality that best approximates the true pattern for the population, given the extensive mortality modelling methods used by the GBD, described elsewhere [26]. For the purposes of comparison of the mortality estimates where the GBD life table is used as the standard, another reliable standard, such as the national UN WPP life table, can also be used.

Two methods to estimate mortality indicators from incomplete death registration using the empirical completeness method and MLTFS are presented in this study: (1) the conventional method and (2) the equivalent deaths method.

#### Conventional method

The conventional method requires an estimate of completeness of registration at ages 5 years and above, which may come from DDMs, the empirical completeness method, or another method. This estimate is used this to adjust the _*45*_*q*_*15*_ upwards, with the adjusted _*45*_*q*_*15*_ and _*5*_*q*_*0*_ inputted into the MLTFS to produce a life table. This is an established method to generate age-specific mortality statistics from incomplete death registration data and is the conventional method described earlier. In our study, we use the empirical completeness method to estimate completeness at all ages. With this all-age completeness estimate, completeness at ages 5 years (${C}_{jk}^{5+}\Big)$ and above is calculated as:

$$ {C}_{jk}^{5+}=\frac{RegD_{jk}^{5+}}{\left(\frac{RegD_{jk}^{All}}{C_{jk}^{All}}-\frac{RegD_{jk}^{<5}}{C_{jk}^{<5}}\right)} $$

where ${RegD}_{jk}^{All}$ is registered deaths at all ages, ${RegD}_{jk}^{5+}$is registered deaths at ages 5 years and above, ${RegD}_{jk}^{<5}$ is registered deaths at ages less than 5 years, ${C}_{jk}^{All}$ is completeness at all ages, ${C}_{jk}^{<5}$ is completeness at ages less than 5 years (used as an input into the empirical completeness method, estimated as the under-five mortality rate from registration data divided by the estimate of the true under-five mortality rate), *j* is country and *k* is calendar year. The denominator is estimated deaths at ages 5 years and above.

The adjusted _*45*_*q*_*15*_ (*Adj*45*q*15_*jk*_) is calculated as:

$$ Adj45q{15}_{jk}=\frac{Reg45q{15}_{jk}}{C_{jk}^{5+}} $$

where *Reg*45*q*15_*jk*_ is _*45*_*q*_*15*_ calculated using registration data.

As mentioned earlier, if the level of completeness at 5 years and above is different from the true completeness for 15-59 years, then the adjusted _*45*_*q*_*15*_ and resultant mortality statistics will be biassed. Furthermore, if the MLT does not reflect the true age pattern of local mortality, this will also bias mortality statistics.

#### Equivalent deaths method

The equivalent deaths method that we propose uses the empirical completeness method and MLTFS to produce the same total number of deaths, by sex, from the following:

- Registered deaths divided by the empirical completeness method’s estimate of completeness (as a fraction).
- The MLTFS’s ${}_n{}{m}_x$values multiplied by the age-specific population.

**Fig. 1:**
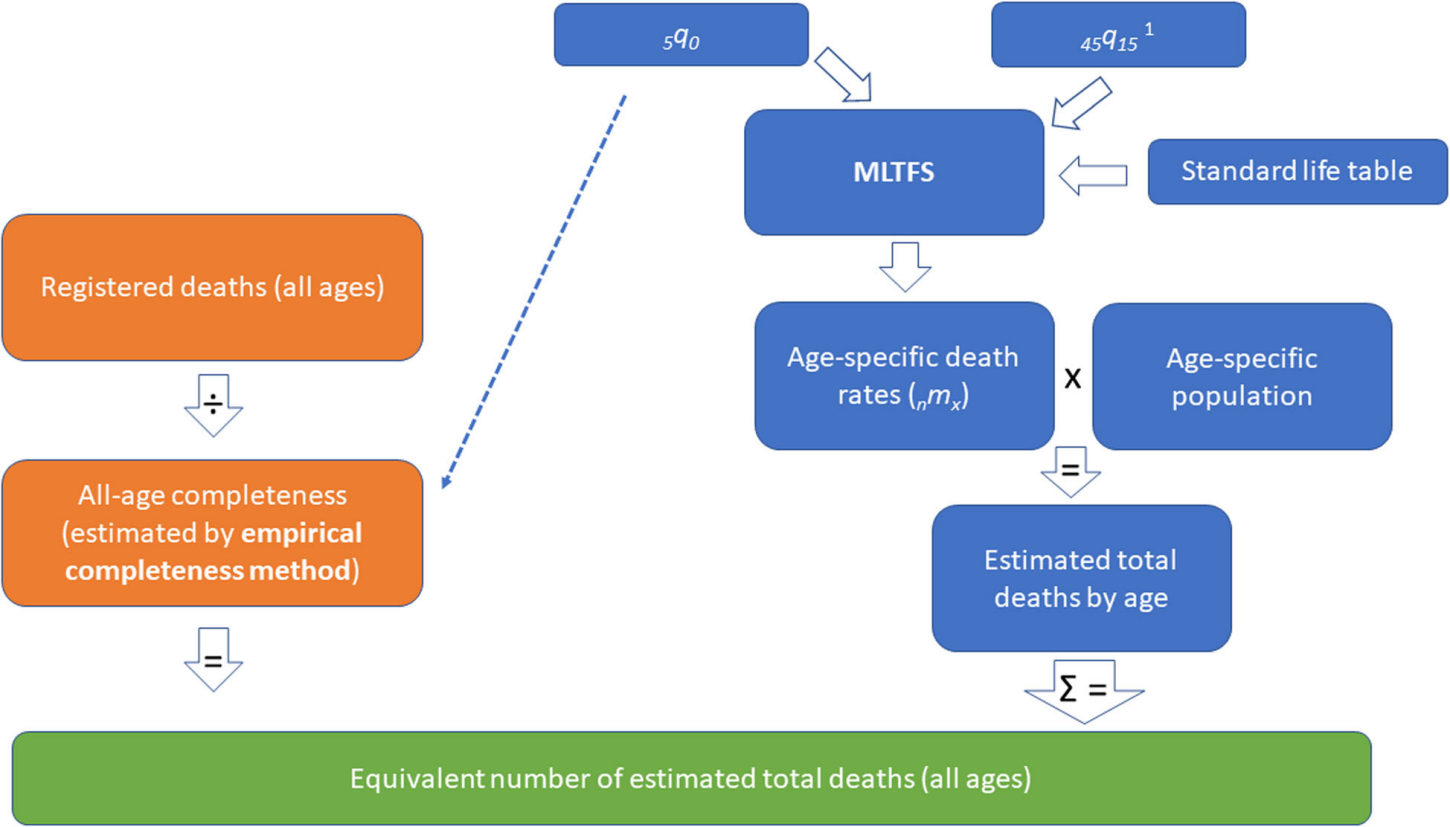
Equivalent deaths method process. ^1^_*45*_*q*_*15*_ is iteratively adjusted until there are an equivalent number of estimated total deaths between the empirical completeness method and MLTFS

If the conventional approach of adjusting _*45*_*q*_*15*_ based on completeness at ages 5 years and above is used, it is highly likely the estimates of total deaths from the empirical completeness method and MLTFS would be different. The equivalent deaths method overcomes this issue—its process is shown in Fig. 1. Only limited data inputs are required:

- Estimated completeness of death registration at all ages, by sex (from the empirical completeness method)
- _5_*q*_0_, by sex, which is the same as for the empirical completeness method
- Number of registered deaths, by age and sex (although age is not necessary for the equivalent deaths method used with model 2)
- Population by age and sex
- MLTFS model coefficients and the GBD national life table (by sex), which is used as a standard life table for national and subnational populations. As mentioned, the GBD national life table is recommended as the standard. The MLTFS coefficients are presented in Additional file 1: Table A.1.

The last remaining input into the MLTFS is the _*45*_*q*_*15*_, which is iteratively adjusted until the total number of deaths by sex according to the MLTFS equals the total number of deaths, by sex, according to the empirical completeness method. In combination with the _*5*_*q*_*0*_ and other inputs, there is only one combination of _*45*_*q*_*15*_ and estimated total deaths. The resultant outputs are ${}_n{}{q}_x$ and ${}_n{}{m}_x$by sex, which can be used to produce summary mortality indicators.

A limitation of the equivalent deaths method is that both age-specific completeness and resultant mortality statistics are reliant on the accuracy of the age pattern of mortality according to the standard life table used and the resultant age pattern of mortality produced by the MLTFS (as with the conventional method). For example, a standard that, compared with the GBD life table, assumes a relatively higher level of adult compared with old-age mortality, would lead to higher estimated _*45*_*q*_*15*_ and lower completeness of registration in ages 15-59 years when applied to the same number of registered deaths and estimated level of all-age completeness. Another limitation is that if there is population age misreporting, as is commonly found in many countries with incomplete death registration data, this will bias estimates of total deaths. Population age misreporting is most likely found at old ages, where death rates are highest. Other sources of error related to both the conventional and equivalent death methods may be from the estimate of all-age completeness (due to, for example, uncertainty in the estimate of the true _*5*_*q*_*0*_) and death registration data (reporting of age at death, date of death or place of residence of the decedent).

### Comparison with capture-recapture studies and alternative mortality estimates from Brazilian states

We firstly compare the two approaches using results from a C-RC study in Brazil, because it provides independent and direct source information of mortality indicators and age-specific completeness that do not rely on MLTs [14]. That C-RC study matched registered deaths to deaths reported in the Ministry of Health (MoH) Mortality Information System (SIM), an independent source. In that C-RC study, total deaths, all-age completeness of registration (i.e. the civil registry) and mortality indicators were estimated by state, sex and age using generalised linear modelling with a series of covariates of demographic, geographic and socio-economic factors, as well as cause of death and place of death [14]. In this study, we compare estimated total deaths, _*45*_*q*_*15*_ and _*25*_*q*_*60*_ (probability of dying between age 60 and 85 years) of the conventional and equivalent death methods for all 27 states with the results in the C-RC study, as well as age-specific completeness for three states with the lowest levels of all-age completeness (Pará, Amapá and Maranhão). Our estimates are made using death registration data, _*5*_*q*_*0*_ estimated by the GBD (which is available for Brazilian states), and the MLTFS with the GBD and, for the purposes of comparison, also the UNWPP life table for Brazil as the standard; the GBD produces life tables for Brazilian states, however, we wanted to explore the utility of the GBD and UNWPP national life tables at the subnational level for the majority of countries where the GBD does not estimate subnational life tables. The comparison of the mortality indicators for all states according to the conventional and equivalent death methods and the estimates of the C-RC study were summarised using the root mean squared difference (RMSD), relative difference (RMSD divided by the average indicator for all states), mean bias and the number of states where the indicator of each method was closer to the C-RC study than the other.

We also compare our results for 2015 to those of three alternative estimates of male _*45*_*q*_*15*_ from Brazil reported in a recent comparative analysis of Brazilian mortality estimates, according to Queiroz et al. for 2000-2010, Schmertmann and Gonzaga for 2010, and the Instituto Brasileiro de Geografia e Estatística (IBGE—Brazil’s national statistics office) for 2000-2010 [27–29]. Queiroz et al. estimated intercensal (2000-2010) completeness of death registration using three death distribution methods, and used completeness to adjust adult mortality, a method consistent with what we describe as the conventional method [28]. Schmertmann and Gonzaga’s Bayesian method combines prior estimates of completeness with a relational model life table [29, 30]. The IBGE estimates of completeness are made using the generalised growth balance method, a form of DDM [28]. Given the different time periods for which the estimates refer to, the most appropriate method of comparison is the correlation coefficient.

### Comparison with capture-recapture studies from Thailand, Vietnam and Oman

Data are also available from C-RC studies in Thailand, Vietnam and Oman [15, 18, 20]. These studies also matched death registration to deaths reported in independent sources; a household survey (Thailand), other routine mortality reporting sources (Vietnam), and deaths reported in a census (Oman). Age-specific completeness in the Thailand, Vietnam and Oman (and sex-specific in Thailand and Vietnam) studies was calculated using the Chandrasekar-Deming method [3]. Mortality indicators were not published with these studies. For each of these studies, we compare all-age completeness and age-specific completeness with the two empirical completeness method applications. Levels of age-specific completeness can be used to generate mortality statistics. Our estimates are made using the published death registration (or reporting) data in these studies, _*5*_*q*_*0*_ estimated by the GBD, and the MLTFS with the GBD life table for the country as the standard.

## Results

### Comparison with capture-recapture studies and alternative mortality estimates from Brazil

Additional File 1: Table A.2 shows that the all-age completeness of the civil registry for Brazilian states according to the empirical completeness method and the C-RC results are very similar, being within 3 percentage points in most states and the largest difference being 7 percentage points for Acre males. In most states, the _*5*_*q*_*0*_ was higher according to the GBD than C-RC.

Table 1 provides summary comparison metrics for mortality indicators of the equivalent deaths and conventional methods (using GBD LT as the standard) versus the C-RC results (state-level results shown in Additional file 1: Tables A.3-A.4). Additional files 2, 3, 4 and 5 demonstrate the application of the equivalent deaths and conventional methods using both the GBD and UNWPP standard life tables. For _*45*_*q*_*15*_, both methods provided estimates very close to C-RC, with the relative difference for the conventional method only 2% for males and 4% for females, and for the equivalent deaths method 5% for males and 7% for females, but this was inflated by the large difference in Maranhão where results were 20% different for females. The mean bias indicated no systematic bias in estimates, whilst the conventional method produced _*45*_*q*_*15*_ estimates closer to C-RC than the equivalent deaths method in 25 of the 27 states for males and 24 out of 27 for females. For _*25*_*q*_*60*_, the relative difference for the equivalent deaths method was 3% for males and for females, and the conventional method 4% for males and for females. The equivalent deaths method provided results closer to C-RC compared with the conventional method in 21 of 27 states for males and 16 of 27 for females. Again, Maranhão was an outlier, adversely affecting the overall results for the conventional method. Of the other states with low completeness, neither the equivalent deaths method nor conventional method was clearly closer to the C-RC study for _*45*_*q*_*15*_ and _*25*_*q*_*60*_, except that the conventional method was much closer for females _*25*_*q*_*60*_ in Amapa.

**Table 1:** Summary comparison metrics compared with C-RC study, Brazil, 2015 [14]

**State**	**Male**_***45***_***q***_***15***_		**Female**_***45***_***q***_***15***_		**Male**_***25***_***q***_***60***_		**Female**_***25***_***q***_***60***_	
	**Equiv.**	**Conv.**	**Equiv.**	**Conv.**	**Equiv.**	**Conv.**	**Equiv.**	**Conv.**
**RMSD**	0.010	0.004	0.007	0.004	0.016	0.027	0.013	0.018
**Relative difference**	5.2%	2.3%	7.2%	4.0%	2.5%	4.1%	2.5%	3.5%
**Mean bias**	+0.001	+0.001	+0.003	+0.003	+0.007	+0.007	+0.003	+0.003
**States closer to C-RC**	2	25	3	24	21	6	16	11
**State**	**Male LE**		**Female LE**		**Male total deaths**		**Female total deaths**	
	**Equiv.**	**Conv.**	**Equiv.**	**Conv.**	**Equiv.**	**Conv.**	**Equiv.**	**Conv.**
**RMSD**	0.51	0.61	0.41	0.61	1002	1419	834	1114
**Relative difference**	0.7%	0.8%	0.5%	0.8%	3.7%	8.9%	4.0%	9.9%
**Mean bias**	−0.18	−0.15	−0.16	−0.18	+75	−286	+122	+29
**States closer to C-RC**	12	15	16	11	14	13	16	11

*LE* life expectancy, *Equiv.* equivalent deaths method, *Conv.* conventional method

Life expectancy estimates of each method were on average within 1 year of the C-RC. The equivalent deaths method provided a smaller RMSD and relative difference than the conventional method, but the conventional method results was closer to C-RC in 15 of 27 states for males and 11 states for females. The overall figures for the conventional method were affected by the large difference with the C-RC in Maranhão of 1.7 years for males and 2.8 years for females, whilst Maranhão’s equivalent deaths method result was almost the same as the C-RC. The relative difference in total deaths for the equivalent deaths method (4% males and females) was less than half that of the conventional method (9% males, 10% females), but these figures were affected by the conventional method performing poorer in states with a higher number of deaths. The equivalent deaths method was closer to C-RC for slightly more states (14 out of 27 states for males, 16 states for females). Overall, the summary comparison metrics when using the UNWPP life table as the standard were very similar to when using the GBD life table (Additional file 1: Tables A.5-A.6). The most notable difference was with the life expectancy for males according to the conventional method, which exhibited a higher RMSD (0.72 UNWPP, 0.61 GBD) and larger negative mean bias (−0.59 UNWPP, −0.15 GBD) when using the UNWPP life table.

Additional file 1: Figure A.1 presents the age-specific completeness of registration according to the C-RC results, the conventional method and equivalent deaths method in three Brazilian states with the lowest levels of completeness (Pará, Amapá and Maranhão). In each state, the C-RC results show completeness was lowest at the youngest ages and also declined with older age, with these trends strongest in Maranhão, where all-age completeness is lowest. Variation in completeness by 5-year age group was greater in the conventional and equivalent deaths methods than the C-RC, exceeding 100% in some age groups. Difference in age-specific completeness between the methods was greatest in Maranhão, whilst there was very little difference between the conventional and equivalent deaths methods in Pará and Amapá. It is worth noting that for these three state differences in mortality statistics for broader age groups shown in Additional file 1: Tables A.3-A.6 are generally smaller than for completeness for 5-year age groups. Age-specific completeness in all 11 states with all-age completeness less than 90% according to the C-RC study is shown in Additional file 1: Table A.7—as expected, age-specific completeness is more constant where all-age completeness is higher.

Comparison of the male _*45*_*q*_*15*_ results from this study with alternative mortality estimates from Brazil show that the average correlation coefficient among our study’s results (equivalent deaths method using GBD and UNWPP life table, conventional method using UNWPP life table) ranged from 0.63 to 0.66, and was more concordant than Queiroz (0.42) and IBGE (0.40) (Table 2, Additional file 1: Table A.8). Only the Bayesian estimates had a similar average correlation coefficient (0.63).

**Table 2:** Correlation coefficient of male _*45*_*q*_*15*_ according to study methods, C-RC and alternative estimates [28]

	Equiv. (GBD std. LT) 2015	Conv. (GBD std. LT) 2015	Equiv. (UN std. LT) 2015	C-RC 2015	Queiroz 2000-2010	IBGE 2000-2010	Bayesian 2010
Queiroz 2000-2010	0.619**	0.442*	0.629**	0.423*		0.188	0.649**
IBGE 2000-2010	0.589**	0.759**	0.585**	0.720**	0.188		0.619**
Bayesian 2010	0.689**	0.771**	0.677**	0.800**	0.649**	0.619**	
**Average**	**0.632**	**0.657**	**0.630**	**0.648**	**0.418**	**0.403**	**0.634**

*Equiv.* equivalent deaths method, *Conv.* conventional method, *Std. LT* standard life table

*p < 0.05

**p < 0.01

### Comparison with capture-recapture studies from Thailand, Vietnam and Oman

In Thailand, at all ages, both methods provided completeness estimates similar to C-RC; the conventional method was closer to C-RC for males, whilst for females the two methods had a similar difference from C-RC. Completeness according to the conventional method was closer to C-RC at ages 15-59 and 60-74 years; however, the equivalent deaths method was closer at 75+ years (Table 3).

**Table 3:** Age-specific completeness of death registration, equivalent deaths method, conventional method and C-RC, by sex, Thailand C-RC study 2005-06 and Vietnam C-RC study 2009 [18, 20]

Country, age	Males			Females		
	Equivalent deaths	Conventional	C-RC	Equivalent deaths	Conventional	C-RC
***Thailand***						
0-14 (both sexes)	85.3	84.8	59.8			
15-59	98.2	93.1	90.9	98.4	92.6	85.2
60-74	100.1	95.6	92.5	100.8	95.4	92.3
75+	84.0	81.3	94.4	87.0	83.4	93.9
All ages	94.2	90.1	91.0	93.9	89.3	91.6
***Vietnam***						
15-59	86.2	81.0	80.4	87.4	80.9	80.5
60-74	69.9	66.2	82.0	75.8	70.5	83.0
75+	88.2	84.9	81.7	83.3	78.9	81.0
All ages	79.3	75.5	81.2	79.8	75.1	81.3

Note: Results for 0-14 years not reported in Vietnam C-RC study

In Vietnam, the equivalent deaths method had a closer all-age completeness to C-RC (2 percentage points) than the conventional method (6 percentage points) (Table 4). Completeness according to the conventional method was however closer to C-RC at 15-59 years and, for males, 75+ years, whilst the equivalent deaths method was closer at 60-74 years for each sex. The conventional method was however substantially different to C-RC at 60-74 years, which caused the greater difference at all ages than the equivalent deaths method.

**Table 4:** Age-specific completeness of death registration, equivalent deaths method, conventional method and C-RC, Oman C-RC study 2010 [15]

Age	Equivalent deaths	Conventional	C-RC
0-14	94.2	94.4	70.0
15-44	64.0	72.3	90.0
45-64	77.7	84.8	97.0
65-84	96.6	100.6	97.0
85+	92.6	91.2	91.0
All ages	86.5	91.5	89.6

In Oman, the conventional method was closer to C-RC at all ages (2 percentage points compared with 5 percentage points for the equivalent deaths method), 15-44, 45-64 and 85+ years, whilst the equivalent deaths method was only closer at 65-84 years (Table 4).

## Discussion

This study has compared the application of two approaches—the conventional and equivalent deaths methods—to estimate mortality indicators from incomplete mortality data that use the empirical completeness method. The methods presented have significant appeal in enabling generation of mortality statistics from limited data, especially at the subnational level where comparator estimates from the GBD and UNWPP are mostly not available. The use of the empirical completeness method to generate mortality indicators from incomplete registration data is supported by it showing very similar levels of all-age completeness to the C-RC studies from the different countries.

The comparison was conducted with C-RC studies, the only independent estimates of age-specific mortality and completeness in populations with incomplete mortality data which are not reliant on assumed age patterns from MLTs. Overall, the conventional and equivalent deaths methods provided estimates of mortality indicators or age-specific completeness mostly concordant with the C-RC studies, with very few exceptions. Estimates of _*45*_*q*_*15*_ and _*25*_*q*_*60*_ in Brazilian states were on average within 5% of the C-RC studies, except for female _*45*_*q*_*15*_ which was significantly inflated by the outlier of Maranhão. Compared to all the C-RC studies, the conventional method overwhelmingly provided closer estimates at 15-59 years (or _*45*_*q*_*15*_), the equivalent deaths method was moderately (but not overwhelmingly) closer for 60-84 years (or _*25*_*q*_*60*_), whilst at all ages (or life expectancy) there was very little difference, with each method being closer for a similar number of populations. The similarity at all ages was due to the larger proportion of deaths and greater impact of life expectancy at 60-84 years than 15-59 years. The use of an alternate standard age pattern of mortality (the UN WPP national life table) made only small difference to results. Also, the male _*45*_*q*_*15*_ findings from the equivalent deaths method (using either GBD or UN WPP standard life table) and conventional method were, on average, more concordant with alternative estimates from Brazil than those made by Queiroz et al. or the IBGE, despite the large time difference between the reference period of each study. These differences in mortality statistics are likely due to the different measurement of completeness of registration used; an advantage of the empirical completeness method is that it can estimate completeness of the most recent year of data, rather than the most recent intercensal period.

A notable characteristic of the C-RC results is that age-specific completeness, above age 5 years, is relatively constant. This may be an artefact of the all-age completeness in these C-RC study populations being over 80%, with the exception of Maranhão, and hence age-specific completeness being less likely to vary by age than if all-age completeness were lower. This characteristic explains why the conventional method provides closer estimates to C-RC studies than the equivalent deaths method at 15-59 years, because it adjusts deaths at ages 15-59 years by the level of completeness at 5 years and above. The closer estimates of the equivalent deaths method to C-RC studies at ages 60-84 years may be because both methods use the same MLT and standard life table, which at those ages may offset differences at 15-59 years for the equivalent deaths method but lead to greater differences for the conventional method. For Maranhão, where all-age completeness is below 80%, age-specific completeness declines at old ages and the national standard life table did not fit its age pattern of mortality well. However, in a setting with lower completeness of registration, poor quality age reporting for mortality and population may affect results.

In the three Brazilian states with the lowest completeness, both methods found that completeness exceeded 100% in some 5-year age groups, which shows the impact of the assumed MLT age pattern in smaller age groups. This may not necessarily be problematic for the production of mortality statistics, because age-specific completeness in excess of 100% occurred in Pará and Amapá but all their mortality statistics at broader age groups were close to that of the C-RC. However, should age-specific completeness greater than 100% occur, users should assess whether there is age misreporting in death or population data (e.g. ‘heaping’ of ages on digits ending in 5 or 10), whether deaths in subnational areas use consistent definitions of residence for deaths and population and whether data inputs into the empirical completeness method are appropriate; users can also potentially use an alternative age standard for the MLT.

The choice of whether to use the equivalent deaths method or conventional method may be best informed by the mortality indicators of interest. From the evidence presented of these populations with relatively high completeness, the conventional method is better at estimating _*45*_*q*_*15*_, the equivalent deaths method is better at estimating _*25*_*q*_*60*_ in most populations, and there is little impact of this choice on life expectancy. In all settings, the accuracy of the MLT and standard life table used will be important in determining accuracy of mortality statistics. Another consideration is that the equivalent deaths method provides the same number of total deaths as that estimated by the empirical completeness method, should this be deemed important; however, this may result in distorted age-specific mortality indicators if population age reporting is incorrect, a common occurrence. Unfortunately, a wider array of C-RC studies, including in lower completeness settings, is not available to further understand how age-specific completeness varies. C-RC study results are themselves subject to limitations which may bias their age-specific accuracy, especially if the assumption of the independence of sources is violated. In the Brazil study, however, the generalised linear models used included several covariates that reduced any bias from source dependency [14].

MLTs make assumptions about the age pattern of mortality that are not always accurate, even in our examples where the GBD country life table was the standard where differences in all-age completeness with the C-RC studies were small. MLTs are developed primarily from historical life tables from high-income countries, so potentially bias mortality estimates in low- and middle-income countries. MLTs other than the MLTFS could potentially be used, such as the Log-Quad and Brass logit MLTs [23, 24, 31]. However, distinct advantages of the MLTFS are its use of a more local relevant standard than the Brass logit MLT and its greater flexibility than the Log-Quad method. A final issue of our analysis is that it did not calculate uncertainty intervals around the estimates of mortality statistics, which would provide additional information with which to assess validity of individual estimates.

Improved completeness of registration and general strengthening of CRVS should increase knowledge of true age patterns of mortality, and how it varies by level of mortality and other important characteristics. Efforts to strengthen death registration systems need to focus not only on increasing the number of registered deaths but also on quality of age reporting of deaths, which is poor in some populations despite the UN Principles and Recommendations recommending the collection of date of birth and date of death, from which to calculate age at death [32]. In India, for example, 34% of registered deaths, or 2.1 million annually, do not report age in publications, thus greatly reducing their utility for guiding public policy [33]. Some countries also do not collect information on the date of birth of the deceased, relying on age reported simply in years, which causes ‘age heaping’, typically at ages ending in a 0 or 5, and more generally poor quality age data. An advantage of the equivalent deaths method is that it can be used, unlike the conventional death method, even without age at death data.

## Conclusions

We have assessed relatively straightforward approaches—the equivalent deaths and conventional methods—that are used with the empirical completeness method to enable analysts to generate mortality statistics from incomplete death registration data. These approaches can be used on registration data as well as other mortality data sources (e.g. Census and HDSS) and only require limited data that are readily available at the national and subnational level.

## Supplementary Information

**Additional File 1: Table A.1.** MLTFS coefficients. **Table A.2.** All-age completeness of Brazil civil registry (empirical completeness method and C-RC) and male and female _*5*_*q*_*0*_ estimates (GBD and C-RC), by sex and state, Brazil, 2015. **Table A.3.**_*45*_*q*_*15*_ and _*25*_*q*_*60,*_ equivalent deaths method (GBD std. LT), conventional method (GBD std. LT) and C-RC, by sex and state, and summary comparison metrics, Brazil, 2015. **Table A.4.** Life expectancy and total deaths, equivalent deaths method (GBD std. LT), conventional method (GBD std. LT) and C-RC, by sex and state, and summary comparison metrics, Brazil, 2015. **Table A.5.**_*45*_*q*_*15*_ and _*25*_*q*_*60,*_ equivalent deaths method (UN WPP std. LT), conventional method (UN WPP std. LT) and C-RC, by sex and state, and summary comparison metrics, Brazil, 2015. **Table A.6.** Life expectancy, equivalent deaths method (UN WPP std. LT), conventional method (UN WPP std. LT) and C-RC, by sex and state, and summary comparison metrics, Brazil, 2015. **Table A.7.** Age-specific completeness of Brazil civil registry (%), by state and sex, 2015, C-RC study. **Table A.8.** Male _*45*_*q*_*15*_ according to equivalent deaths method (GBD and UN std. LT), conventional method (GBD std. LT), C-RC, Queiroz, IBGE and Bayesian. **Figure A.1.** Age-specific completeness of Brazil civil registry (%), equivalent deaths method (GBD std. LT), conventional method (GBD std. LT) and C-RC, by sex, Pará, Amapá and Maranhão, 2015.

**Additional File 2.** Demonstration of Equivalent deaths method (GBD standard life table) in Brazilian states, 2015.

**Additional File 3.** Demonstration of Conventional deaths method (GBD standard life table) in Brazilian states, 2015.

**Additional File 4.** Demonstration of Equivalent deaths method (UN standard life table) in Brazilian states, 2015.

**Additional File 5.** Demonstration of Conventional deaths method (UN standard life table) in Brazilian states, 2015.

## Data Availability

The data from the Brazil, Thailand, Oman and Vietnam studies is shown in their respective references. The demonstration of the equivalent deaths and conventional methods for Brazil is shown in Additional files 2, 3, 4 and 5.

## Abbreviations

C-RC: Capture-recapture
CRVS: Civil registration and vital statistics system
DDM: Death distribution methods
GBD: Global Burden of Disease
HDSS: Health and Demographic Surveillance System
IBGE: Instituto Brasileiro de Geografia e Estatística
IGME: International Group for Mortality Estimation
MLT: Model life table
MLTFS: Model life table with flexible standards
MoH: Ministry of Health
RMSD: Root mean squared difference
SDG: Sustainable Development Goals
SIM: Mortality Information System
UNWPP: United Nations World Population Prospects
