# Supplementary material for: Generating age-specific mortality statistics from incomplete death registration data: two applications of the empirical completeness method
Source: Popul Health Metr. 2021 Jun 7;19:29. doi: 10.1186/s12963-021-00262-3 (PMC8186206; doi:10.1186/s12963-021-00262-3)
Supplement: Supplementary file 1 — Additional File 1: Table A.1. MLTFS coefficients. Table A.2. All-age completeness of Brazil civil registry (empirical completeness method and C-RC) and male and female 5q0 estimates (GBD and C-RC), by sex and state, Brazil, 2015. Table A.3. 45q15 and 25q60, equivalent deaths method (GBD std. LT), conventional method (GBD std. LT) and C-RC, by sex and state, and summary comparison metrics, Brazil, 2015. Table A.4. Life expectancy and total deaths, equivalent deaths method (GBD std. LT), conventional method (GBD std. LT) and C-RC, by sex and state, and summary comparison metrics, Brazil, 2015. Table A.5. 45q15 and 25q60, equivalent deaths method (UN WPP std. LT), conventional method (UN WPP std. LT) and C-RC, by sex and state, and summary comparison metrics, Brazil, 2015. Table A.6. Life expectancy, equivalent deaths method (UN WPP std. LT), conventional method (UN WPP std. LT) and C-RC, by sex and state, and summary comparison metrics, Brazil, 2015. Table A.7. Age-specific completeness of Brazil civil registry (%), by state and sex, 2015, C-RC study. Table A.8. Male 45q15 according to equivalent deaths method (GBD and UN std. LT), conventional method (GBD std. LT), C-RC, Queiroz, IBGE and Bayesian. Figure A.1. Age-specific completeness of Brazil civil registry (%), equivalent deaths method (GBD std. LT), conventional method (GBD std. LT) and C-RC, by sex, Pará, Amapá and Maranhão, 2015. [file 12963_2021_262_MOESM1_ESM.docx]

**Additional File 1: Additional tables and figure**

**Table A.1: MLTFS coefficients [1]**

|  | **Males** | | **Females** | |
| --- | --- | --- | --- | --- |
| ***Age (x)*** | **β_1_** | **β_2_** | **β_1_** | **β_2_** |
| 0 | 0.993 |  | 0.982 |  |
| 1-4 | 1.005 |  | 1.047 |  |
| 5-9 | 0.823 |  | 0.766 |  |
| 10-14 | 0.468 | 0.386 | 0.368 | 0.507 |
| 15-19 | 0.134 | 0.823 | 0.147 | 0.776 |
| 20-24 | 0.042 | 0.828 | 0.136 | 0.865 |
| 25-29 | 0.029 | 0.775 | 0.121 | 0.925 |
| 30-34 | 0.018 | 0.784 | 0.094 | 0.935 |
| 35-39 |  | 0.871 |  | 1.064 |
| 40-44 |  | 0.906 |  | 0.990 |
| 45-49 |  | 0.924 |  | 0.921 |
| 50-54 |  | 0.903 |  | 0.878 |
| 55-59 |  | 0.855 |  | 0.860 |
| 60-64 |  | 0.792 |  | 0.838 |
| 65-69 |  | 0.786 |  | 0.866 |
| 70-74 |  | 0.801 |  | 0.911 |
| 75-79 |  | 0.799 |  | 0.914 |
| 80-84 |  | 0.709 |  | 0.832 |

**Table A.2: All-age completeness of Brazil civil registry (empirical completeness method and C-RC) and male and female *_5_q_0_* estimates (GBD and C-RC), by sex and state, Brazil, 2015 [2]**

|  | **Male completeness (%)** | | **Female completeness (%)** | | **Male *_5_q_0_*** | | **Female *_5_q_0_*** | |
| --- | --- | --- | --- | --- | --- | --- | --- | --- |
| **State** | **Empir.** | **C-RC** | **Empir.** | **C-RC** | **GBD** | **C-RC** | **GBD** | **C-RC** |
| Acre | 87.1 | 94.1 | 90.8 | 94.5 | 0.029 | 0.021 | 0.023 | 0.020 |
| Amazonas | 91.3 | 89.2 | 91.9 | 89.6 | 0.021 | 0.019 | 0.017 | 0.017 |
| Roraima | 88.6 | 90.7 | 85.6 | 90.5 | 0.026 | 0.023 | 0.021 | 0.018 |
| Pará | 87.0 | 82.1 | 86.3 | 82.5 | 0.021 | 0.019 | 0.017 | 0.016 |
| Amapá | 85.1 | 80.2 | 84.6 | 82.6 | 0.024 | 0.021 | 0.020 | 0.017 |
| Tocantins | 89.0 | 90.5 | 88.9 | 89.3 | 0.023 | 0.017 | 0.018 | 0.014 |
| Maranhão | 75.4 | 75.7 | 70.0 | 68.0 | 0.027 | 0.019 | 0.022 | 0.016 |
| Piauí | 85.2 | 88.6 | 82.8 | 86.5 | 0.025 | 0.019 | 0.020 | 0.015 |
| Ceará | 89.9 | 92.2 | 88.6 | 92.8 | 0.024 | 0.015 | 0.019 | 0.013 |
| Rio Grande do Norte | 94.3 | 89.7 | 93.5 | 87.3 | 0.014 | 0.016 | 0.011 | 0.015 |
| Bahia | 90.4 | 92.1 | 89.8 | 91.7 | 0.024 | 0.018 | 0.020 | 0.016 |
| Rondônia | 97.1 | 98.0 | 96.8 | 97.6 | 0.017 | 0.018 | 0.014 | 0.014 |
| Paraíba | 95.1 | 93.3 | 94.1 | 92.7 | 0.019 | 0.016 | 0.015 | 0.012 |
| Pernambuco | 93.0 | 95.7 | 93.4 | 96.3 | 0.023 | 0.016 | 0.018 | 0.014 |
| Alagoas | 94.7 | 91.6 | 93.6 | 91.0 | 0.021 | 0.019 | 0.016 | 0.015 |
| Sergipe | 92.6 | 92.5 | 92.3 | 91.1 | 0.023 | 0.018 | 0.019 | 0.018 |
| Minas Gerais | 94.8 | 98.0 | 94.6 | 97.2 | 0.018 | 0.014 | 0.015 | 0.012 |
| Espírito Santo | 95.9 | 97.2 | 94.8 | 97.2 | 0.018 | 0.014 | 0.014 | 0.012 |
| Rio de Janeiro | 97.2 | 98.6 | 97.3 | 98.4 | 0.018 | 0.016 | 0.014 | 0.014 |
| São Paulo | 97.0 | 99.6 | 96.8 | 9.4 | 0.015 | 0.014 | 0.012 | 0.011 |
| Paraná | 96.6 | 98.7 | 96.6 | 98.3 | 0.016 | 0.013 | 0.013 | 0.012 |
| Santa Catarina | 92.2 | 98.1 | 91.5 | 96.7 | 0.019 | 0.013 | 0.015 | 0.010 |
| Rio Grande do Sul | 97.6 | 99.4 | 97.6 | 99.4 | 0.014 | 0.013 | 0.011 | 0.011 |
| Mato Grosso do Sul | 97.9 | 97.2 | 97.7 | 96.8 | 0.014 | 0.016 | 0.012 | 0.013 |
| Mato Grosso | 96.9 | 93.9 | 97.0 | 94.1 | 0.016 | 0.018 | 0.013 | 0.017 |
| Goiás | 94.7 | 95.5 | 94.0 | 96.1 | 0.019 | 0.015 | 0.016 | 0.013 |
| Distriro Federal | 94.1 | 99.0 | 95.0 | 98.2 | 0.016 | 0.014 | 0.013 | 0.013 |

Empir.: Empirical completeness method

**Table A.3: *_45_q_15_* and *_25_q_60,_* equivalent deaths method (GBD std. LT), conventional method (GBD std. LT) and C-RC, by sex and state, and summary comparison metrics, Brazil, 2015 [2]**

|  | **Male *_45_q_15_*** | | | **Female *_45_q_15_*** | | | **Male *_25_q_60_*** | | | **Female *_25_q_60_*** | | |
| --- | --- | --- | --- | --- | --- | --- | --- | --- | --- | --- | --- | --- |
| **State** | **Equiv.** | **Conv.** | **C-RC** | **Equiv.** | **Conv.** | **C-RC** | **Equiv.** | **Conv.** | **C-RC** | **Equiv.** | **Conv.** | **C-RC** |
| **Completeness <90%** | | | | | | | | | | | | |
| Acre | 0.198 | 0.197 | 0.185 | 0.099 | 0.095 | 0.091 | 0.671 | 0.671 | 0.663 | 0.544 | 0.533 | 0.539 |
| Amazonas | 0.195 | 0.187 | 0.188 | 0.095 | 0.093 | 0.093 | 0.668 | 0.654 | 0.682 | 0.532 | 0.525 | 0.539 |
| Roraima | 0.202 | 0.201 | 0.197 | 0.102 | 0.093 | 0.089 | 0.678 | 0.677 | 0.664 | 0.554 | 0.524 | 0.531 |
| Pará | 0.186 | 0.184 | 0.191 | 0.087 | 0.089 | 0.091 | 0.653 | 0.650 | 0.667 | 0.506 | 0.512 | 0.529 |
| Amapá | 0.184 | 0.186 | 0.197 | 0.087 | 0.095 | 0.095 | 0.650 | 0.653 | 0.672 | 0.506 | 0.532 | 0.533 |
| Tocantins | 0.174 | 0.183 | 0.181 | 0.096 | 0.097 | 0.095 | 0.633 | 0.648 | 0.596 | 0.534 | 0.538 | 0.523 |
| Maranhão | 0.186 | 0.208 | 0.200 | 0.080 | 0.106 | 0.098 | 0.652 | 0.687 | 0.634 | 0.479 | 0.566 | 0.493 |
| Piauí | 0.200 | 0.200 | 0.194 | 0.097 | 0.092 | 0.086 | 0.676 | 0.676 | 0.643 | 0.538 | 0.522 | 0.516 |
| Ceará | 0.203 | 0.208 | 0.207 | 0.096 | 0.089 | 0.085 | 0.680 | 0.687 | 0.634 | 0.535 | 0.512 | 0.514 |
| Rio Grande do Norte | 0.180 | 0.190 | 0.199 | 0.079 | 0.082 | 0.083 | 0.644 | 0.660 | 0.624 | 0.477 | 0.487 | 0.480 |
| Bahia | 0.197 | 0.211 | 0.208 | 0.090 | 0.098 | 0.094 | 0.670 | 0.691 | 0.632 | 0.514 | 0.541 | 0.494 |
| **Completeness >90%** | | | | | | | | | | | | |
| Rondônia | 0.195 | 0.179 | 0.177 | 0.102 | 0.123 | 0.085 | 0.667 | 0.642 | 0.681 | 0.555 | 0.614 | 0.573 |
| Paraíba | 0.200 | 0.205 | 0.210 | 0.097 | 0.093 | 0.093 | 0.675 | 0.683 | 0.664 | 0.539 | 0.525 | 0.520 |
| Pernambuco | 0.229 | 0.224 | 0.219 | 0.108 | 0.100 | 0.097 | 0.716 | 0.710 | 0.700 | 0.572 | 0.547 | 0.569 |
| Alagoas | 0.228 | 0.232 | 0.235 | 0.107 | 0.105 | 0.106 | 0.715 | 0.720 | 0.717 | 0.568 | 0.564 | 0.592 |
| Sergipe | 0.229 | 0.230 | 0.234 | 0.098 | 0.095 | 0.094 | 0.716 | 0.718 | 0.690 | 0.540 | 0.531 | 0.544 |
| Minas Gerais | 0.179 | 0.186 | 0.182 | 0.088 | 0.091 | 0.089 | 0.641 | 0.654 | 0.624 | 0.510 | 0.520 | 0.495 |
| Espírito Santo | 0.181 | 0.191 | 0.189 | 0.083 | 0.088 | 0.086 | 0.645 | 0.661 | 0.630 | 0.490 | 0.508 | 0.473 |
| Rio de Janeiro | 0.213 | 0.201 | 0.199 | 0.102 | 0.107 | 0.106 | 0.694 | 0.677 | 0.697 | 0.555 | 0.569 | 0.543 |
| São Paulo | 0.189 | 0.175 | 0.171 | 0.092 | 0.087 | 0.085 | 0.658 | 0.635 | 0.671 | 0.522 | 0.505 | 0.513 |
| Paraná | 0.191 | 0.186 | 0.183 | 0.094 | 0.089 | 0.087 | 0.662 | 0.653 | 0.667 | 0.529 | 0.510 | 0.533 |
| Santa Catarina | 0.177 | 0.162 | 0.155 | 0.087 | 0.082 | 0.078 | 0.638 | 0.612 | 0.661 | 0.505 | 0.488 | 0.492 |
| Rio Grande do Sul | 0.191 | 0.181 | 0.178 | 0.090 | 0.089 | 0.088 | 0.661 | 0.645 | 0.668 | 0.516 | 0.512 | 0.510 |
| Mato Grosso do Sul | 0.188 | 0.187 | 0.188 | 0.095 | 0.096 | 0.096 | 0.657 | 0.654 | 0.658 | 0.533 | 0.535 | 0.528 |
| Mato Grosso | 0.189 | 0.193 | 0.198 | 0.096 | 0.092 | 0.092 | 0.658 | 0.665 | 0.648 | 0.534 | 0.523 | 0.545 |
| Goiás | 0.205 | 0.204 | 0.203 | 0.102 | 0.095 | 0.093 | 0.682 | 0.681 | 0.673 | 0.553 | 0.531 | 0.559 |
| Distriro Federal | 0.167 | 0.155 | 0.149 | 0.082 | 0.076 | 0.073 | 0.620 | 0.599 | 0.623 | 0.487 | 0.464 | 0.470 |
| **RMSD** | **0.010** | **0.004** | **-** | **0.007** | **0.004** | **-** | **0.016** | **0.027** | **-** | **0.013** | **0.018** | **-** |
| **Relative difference** | **5.2%** | **2.3%** | **-** | **7.2%** | **4.0%** | **-** | **2.5%** | **4.1%** | **-** | **2.5%** | **3.5%** | **-** |
| **Mean bias** | **+0.001** | **+0.001** | **-** | **+0.003** | **+0.003** | **-** | **+0.007** | **+0.007** | **-** | **+0.003** | **+0.003** | **-** |
| **States closer to C-RC** | **2** | **25** | **-** | **3** | **24** | **-** | **21** | **6** | **-** | **16** | **11** | **-** |

Equiv.: Equivalent deaths method

Conv.: Conventional method

Std. LT: Standard life table

RMSD: Root mean squared difference

**Table A.4: Life expectancy and total deaths, equivalent deaths method (GBD std. LT), conventional method (GBD std. LT) and C-RC, by sex and state, and summary comparison metrics, Brazil, 2015 [2]**

|  | **Male LE** | | | **Female LE** | | | **Male total deaths** | | | **Female total deaths** | | |
| --- | --- | --- | --- | --- | --- | --- | --- | --- | --- | --- | --- | --- |
| **State** | **Equiv.** | **Conv.** | **C-RC** | **Equiv.** | **Conv.** | **C-RC** | **Equiv.** | **Conv.** | **C-RC** | **Equiv.** | **Conv.** | **C-RC** |
| **Completeness <90%** | | | | | | | | | | | | |
| Acre | 70.5 | 70.5 | 71.9 | 77.6 | 78.0 | 78.1 | 2,402 | 2,399 | 2,222 | 1,516 | 1,476 | 1,457 |
| Amazonas | 71.4 | 72.0 | 71.1 | 78.6 | 78.8 | 78.3 | 10,350 | 9,969 | 10,592 | 6,575 | 6,472 | 6,743 |
| Roraima | 70.6 | 70.6 | 70.9 | 77.8 | 78.8 | 78.3 | 1,412 | 1,408 | 1,379 | 815 | 758 | 771 |
| Pará | 72.0 | 72.1 | 71.2 | 79.4 | 79.2 | 78.9 | 22,954 | 22,780 | 24,320 | 14,375 | 14,607 | 15,037 |
| Amapá | 71.8 | 71.7 | 70.9 | 79.1 | 78.3 | 78.9 | 1,885 | 1,904 | 2,002 | 1,162 | 1,236 | 1,191 |
| Tocantins | 72.7 | 72.0 | 73.0 | 78.5 | 78.3 | 78.6 | 4,739 | 4,941 | 4,660 | 3,089 | 3,120 | 3,075 |
| Maranhão | 71.5 | 70.0 | 71.7 | 79.9 | 77.0 | 79.8 | 21,635 | 23,729 | 21,550 | 14,044 | 17,393 | 14,440 |
| Piauí | 70.7 | 70.7 | 71.6 | 78.1 | 78.6 | 78.8 | 11,836 | 11,836 | 11,379 | 8,949 | 8,590 | 8,574 |
| Ceará | 70.6 | 70.3 | 71.2 | 78.3 | 79.0 | 79.3 | 33,658 | 34,357 | 32,835 | 25,877 | 24,421 | 24,728 |
| Rio Grande do Norte | 72.9 | 72.2 | 71.8 | 80.8 | 80.4 | 79.8 | 11,397 | 11,896 | 11,988 | 8,581 | 8,830 | 9,193 |
| Bahia | 70.9 | 70.0 | 71.2 | 78.9 | 78.0 | 79.3 | 53,501 | 56,680 | 52,519 | 39,969 | 42,862 | 39,107 |
| **Completeness >90%** | | | | | | | | | | | | |
| Rondônia | 71.7 | 72.8 | 71.7 | 78.3 | 76.3 | 78.1 | 5,241 | 4,877 | 5,206 | 3,110 | 3,642 | 3,068 |
| Paraíba | 71.2 | 70.9 | 71.0 | 78.5 | 78.9 | 78.6 | 15,236 | 15,556 | 15,500 | 12,473 | 12,050 | 12,571 |
| Pernambuco | 69.1 | 69.4 | 69.9 | 77.1 | 78.0 | 77.8 | 36,573 | 35,927 | 35,391 | 29,497 | 27,663 | 28,684 |
| Alagoas | 69.3 | 69.1 | 68.9 | 77.4 | 77.5 | 77.1 | 2,402 | 2,398 | 11,885 | 1,516 | 1,476 | 8,912 |
| Sergipe | 69.1 | 69.0 | 69.2 | 78.1 | 78.4 | 78.1 | 8,023 | 8,069 | 8,056 | 5,620 | 5,488 | 5,665 |
| Minas Gerais | 72.7 | 72.2 | 73.3 | 79.5 | 79.2 | 80.1 | 77,577 | 80,302 | 75,311 | 61,220 | 62,922 | 59,211 |
| Espírito Santo | 72.6 | 71.9 | 72.8 | 80.4 | 79.7 | 80.8 | 13,385 | 14,000 | 13,190 | 9,774 | 10,258 | 9,532 |
| Rio de Janeiro | 70.6 | 71.3 | 70.9 | 78.1 | 77.7 | 78.3 | 70,752 | 67,374 | 69,924 | 64,258 | 66,575 | 63,589 |
| São Paulo | 72.4 | 73.3 | 73.1 | 79.5 | 80.1 | 79.9 | 161,169 | 151,165 | 157,175 | 134,394 | 128,447 | 130,762 |
| Paraná | 72.1 | 72.5 | 72.5 | 79.2 | 79.8 | 79.5 | 41,678 | 40,672 | 40,888 | 31,304 | 29,795 | 30,674 |
| Santa Catarina | 72.8 | 73.9 | 74.0 | 79.8 | 80.4 | 80.8 | 23,136 | 21,476 | 21,905 | 17,879 | 17,107 | 16,786 |
| Rio Grande do Sul | 72.3 | 73.0 | 72.8 | 79.7 | 79.9 | 80.0 | 45,684 | 43,616 | 44,891 | 38,998 | 38,635 | 38,268 |
| Mato Grosso do Sul | 72.4 | 72.6 | 72.3 | 79.1 | 79.0 | 78.9 | 9,205 | 9,126 | 9,292 | 6,483 | 6,523 | 6,521 |
| Mato Grosso | 72.2 | 71.9 | 71.6 | 78.9 | 79.3 | 78.4 | 10,680 | 10,883 | 11,012 | 6,421 | 6,228 | 6,579 |
| Goiás | 70.9 | 70.9 | 71.1 | 78.1 | 78.8 | 78.4 | 24,017 | 23,948 | 23,732 | 16,262 | 15,339 | 15,939 |
| Distriro Federal | 74.0 | 74.9 | 74.3 | 80.8 | 81.6 | 80.9 | 7,145 | 6,722 | 6,833 | 5,574 | 5,229 | 5,355 |
| **RMSD** | **0.51** | **0.61** | **-** | **0.41** | **0.61** | **-** | **1,002** | **1,419** | **-** | **834** | **1,114** | **-** |
| **Relative difference** | **0.7%** | **0.8%** | **-** | **0.5%** | **0.8%** | **-** | **3.7%** | **8.9%** | **-** | **4.0%** | **9.9%** | **-** |
| **Mean bias** | **-0.18** | **-0.15** | **-** | **-0.16** | **-0.18** | **-** | **+75** | **-286** | **-** | **+122** | **+29** | **-** |
| **States closer to C-RC** | **12** | **15** | **-** | **16** | **11** | **-** | **14** | **13** | **-** | **16** | **11** | **-** |

LE: Life expectancy

Equiv.: Equivalent deaths method

Conv.: Conventional method

Std. LT: Standard life table

RMSD: Root mean squared difference

**Table A.5: *_45_q_15_* and *_25_q_60,_* equivalent deaths method (UN WPP std. LT), conventional method (UN WPP std. LT) and C-RC, by sex and state, and summary comparison metrics, Brazil, 2015 [2]**

|  | **Male *_45_q_15_*** | | | **Female *_45_q_15_*** | | | **Male *_25_q_60_*** | | | **Female *_25_q_60_*** | | |
| --- | --- | --- | --- | --- | --- | --- | --- | --- | --- | --- | --- | --- |
| **State** | **Equiv.** | **Conv.** | **C-RC** | **Equiv.** | **Conv.** | **C-RC** | **Equiv.** | **Conv.** | **C-RC** | **Equiv.** | **Conv.** | **C-RC** |
| **Completeness <90%** | | | | | | | | | | | | |
| Acre | 0.190 | 0.197 | 0.185 | 0.099 | 0.095 | 0.091 | 0.672 | 0.683 | 0.663 | 0.540 | 0.529 | 0.539 |
| Amazonas | 0.189 | 0.187 | 0.188 | 0.095 | 0.093 | 0.093 | 0.670 | 0.666 | 0.682 | 0.528 | 0.522 | 0.539 |
| Roraima | 0.196 | 0.201 | 0.197 | 0.102 | 0.093 | 0.089 | 0.682 | 0.689 | 0.664 | 0.549 | 0.520 | 0.531 |
| Pará | 0.179 | 0.184 | 0.191 | 0.087 | 0.089 | 0.091 | 0.654 | 0.663 | 0.667 | 0.502 | 0.509 | 0.529 |
| Amapá | 0.178 | 0.186 | 0.197 | 0.087 | 0.095 | 0.095 | 0.652 | 0.665 | 0.672 | 0.502 | 0.529 | 0.533 |
| Tocantins | 0.166 | 0.183 | 0.181 | 0.096 | 0.097 | 0.095 | 0.632 | 0.660 | 0.596 | 0.531 | 0.535 | 0.523 |
| Maranhão | 0.178 | 0.208 | 0.200 | 0.080 | 0.106 | 0.098 | 0.652 | 0.698 | 0.634 | 0.477 | 0.562 | 0.493 |
| Piauí | 0.192 | 0.200 | 0.194 | 0.097 | 0.092 | 0.086 | 0.675 | 0.687 | 0.643 | 0.535 | 0.517 | 0.516 |
| Ceará | 0.194 | 0.208 | 0.207 | 0.097 | 0.089 | 0.085 | 0.678 | 0.699 | 0.634 | 0.533 | 0.509 | 0.514 |
| Rio Grande do Norte | 0.171 | 0.190 | 0.199 | 0.080 | 0.082 | 0.083 | 0.641 | 0.671 | 0.624 | 0.475 | 0.484 | 0.480 |
| Bahia | 0.188 | 0.211 | 0.208 | 0.090 | 0.098 | 0.094 | 0.669 | 0.703 | 0.632 | 0.512 | 0.536 | 0.494 |
| **Completeness >90%** | | | | | | | | | | | | |
| Rondônia | 0.189 | 0.179 | 0.177 | 0.102 | 0.123 | 0.085 | 0.670 | 0.654 | 0.681 | 0.550 | 0.610 | 0.573 |
| Paraíba | 0.190 | 0.205 | 0.210 | 0.098 | 0.093 | 0.093 | 0.672 | 0.695 | 0.664 | 0.537 | 0.521 | 0.520 |
| Pernambuco | 0.220 | 0.224 | 0.219 | 0.109 | 0.100 | 0.097 | 0.716 | 0.721 | 0.700 | 0.570 | 0.543 | 0.569 |
| Alagoas | 0.220 | 0.232 | 0.235 | 0.107 | 0.105 | 0.106 | 0.716 | 0.732 | 0.717 | 0.565 | 0.560 | 0.592 |
| Sergipe | 0.221 | 0.230 | 0.234 | 0.098 | 0.095 | 0.094 | 0.717 | 0.730 | 0.690 | 0.537 | 0.527 | 0.544 |
| Minas Gerais | 0.170 | 0.186 | 0.182 | 0.089 | 0.091 | 0.089 | 0.640 | 0.666 | 0.624 | 0.507 | 0.516 | 0.495 |
| Espírito Santo | 0.173 | 0.191 | 0.189 | 0.083 | 0.088 | 0.086 | 0.644 | 0.673 | 0.630 | 0.488 | 0.504 | 0.473 |
| Rio de Janeiro | 0.205 | 0.201 | 0.199 | 0.103 | 0.107 | 0.106 | 0.694 | 0.689 | 0.697 | 0.553 | 0.565 | 0.543 |
| São Paulo | 0.181 | 0.175 | 0.171 | 0.093 | 0.087 | 0.085 | 0.658 | 0.647 | 0.671 | 0.520 | 0.501 | 0.513 |
| Paraná | 0.184 | 0.186 | 0.183 | 0.094 | 0.089 | 0.087 | 0.662 | 0.665 | 0.667 | 0.526 | 0.506 | 0.533 |
| Santa Catarina | 0.170 | 0.162 | 0.155 | 0.087 | 0.082 | 0.078 | 0.639 | 0.624 | 0.661 | 0.502 | 0.484 | 0.492 |
| Rio Grande do Sul | 0.183 | 0.181 | 0.178 | 0.091 | 0.089 | 0.088 | 0.661 | 0.657 | 0.668 | 0.514 | 0.508 | 0.510 |
| Mato Grosso do Sul | 0.181 | 0.187 | 0.188 | 0.096 | 0.096 | 0.096 | 0.657 | 0.666 | 0.658 | 0.529 | 0.531 | 0.528 |
| Mato Grosso | 0.183 | 0.193 | 0.198 | 0.096 | 0.092 | 0.092 | 0.660 | 0.677 | 0.648 | 0.530 | 0.519 | 0.545 |
| Goiás | 0.197 | 0.204 | 0.203 | 0.102 | 0.095 | 0.093 | 0.683 | 0.693 | 0.673 | 0.549 | 0.527 | 0.559 |
| Distriro Federal | 0.162 | 0.155 | 0.149 | 0.082 | 0.076 | 0.073 | 0.624 | 0.612 | 0.623 | 0.483 | 0.460 | 0.470 |
| **RMSD** | **0.011** | **0.004** | **-** | **0.007** | **0.004** | **-** | **0.016** | **0.029** | **-** | **0.013** | **0.019** | **-** |
| **Relative difference** | **5.8%** | **2.3%** | **-** | **7.2%** | **4.0%** | **-** | **2.4%** | **4.6%** | **-** | **2.5%** | **3.6%** | **-** |
| **Mean bias** | **-0.006** | **+0.001** | **-** | **+0.003** | **+0.003** | **-** | **+0.008** | **+0.019** | **-** | **0.000** | **-0.001** | **-** |
| **States closer to C-RC** | **6** | **21** | **-** | **3** | **24** | **-** | **24** | **3** | **-** | **17** | **10** | **-** |

Equiv.: Equivalent deaths method

Conv.: Conventional method

Std. LT: Standard life table

RMSD: Root mean squared difference

**Table A.6: Life expectancy, equivalent deaths method (UN WPP std. LT), conventional method (UN WPP std. LT) and C-RC, by sex and state, and summary comparison metrics, Brazil, 2015 [2]**

|  | **Male LE** | | | **Female LE** | | | |
| --- | --- | --- | --- | --- | --- | --- | --- |
| **State** | **Equiv.** | **Conv.** | **C-RC** | **Equiv.** | **Conv.** | **C-RC** | |
| **Completeness <90%** | | | | | | |  |
| Acre | 70.6 | 70.1 | 71.9 | 77.7 | 78.0 | 78.1 | |
| Amazonas | 71.3 | 71.5 | 71.1 | 78.6 | 78.8 | 78.3 | |
| Roraima | 70.5 | 70.2 | 70.9 | 77.9 | 78.9 | 78.3 | |
| Pará | 71.9 | 71.6 | 71.2 | 79.5 | 79.2 | 78.9 | |
| Amapá | 71.8 | 71.3 | 70.9 | 79.2 | 78.3 | 78.9 | |
| Tocantins | 72.7 | 71.6 | 73.0 | 78.5 | 78.3 | 78.6 | |
| Maranhão | 71.5 | 69.7 | 71.7 | 79.9 | 77.0 | 79.8 | |
| Piauí | 70.7 | 70.2 | 71.6 | 78.0 | 78.7 | 78.8 | |
| Ceará | 70.7 | 69.9 | 71.2 | 78.2 | 79.0 | 79.3 | |
| Rio Grande do Norte | 73.0 | 71.8 | 71.8 | 80.8 | 80.5 | 79.8 | |
| Bahia | 71.0 | 69.6 | 71.2 | 78.8 | 78.0 | 79.3 | |
| **Completeness >90%** | | | | | | |  |
| Rondônia | 71.7 | 72.3 | 71.7 | 78.4 | 76.3 | 78.1 | |
| Paraíba | 71.4 | 70.4 | 71.0 | 78.4 | 79.0 | 78.6 | |
| Pernambuco | 69.2 | 69.0 | 69.9 | 77.1 | 78.0 | 77.8 | |
| Alagoas | 69.4 | 68.7 | 68.9 | 77.4 | 77.6 | 77.1 | |
| Sergipe | 69.2 | 68.6 | 69.2 | 78.1 | 78.4 | 78.1 | |
| Minas Gerais | 72.7 | 71.7 | 73.3 | 79.5 | 79.2 | 80.1 | |
| Espírito Santo | 72.7 | 71.5 | 72.8 | 80.4 | 79.8 | 80.8 | |
| Rio de Janeiro | 70.7 | 70.9 | 70.9 | 78.1 | 77.7 | 78.3 | |
| São Paulo | 72.4 | 72.8 | 73.1 | 79.5 | 80.1 | 79.9 | |
| Paraná | 72.2 | 72.0 | 72.5 | 79.2 | 79.9 | 79.5 | |
| Santa Catarina | 72.8 | 73.4 | 74.0 | 79.8 | 80.4 | 80.8 | |
| Rio Grande do Sul | 72.4 | 72.5 | 72.8 | 79.7 | 79.9 | 80.0 | |
| Mato Grosso do Sul | 72.5 | 72.1 | 72.3 | 79.1 | 79.1 | 78.9 | |
| Mato Grosso | 72.2 | 71.5 | 71.6 | 79.0 | 79.4 | 78.4 | |
| Goiás | 70.9 | 70.5 | 71.1 | 78.1 | 78.9 | 78.4 | |
| Distriro Federal | 73.8 | 74.3 | 74.3 | 80.8 | 81.7 | 80.9 | |
| **RMSD** | **0.49** | **0.72** | **-** | **0.43** | **0.62** | **-** | |
| **Relative difference** | **0.7%** | **1.0%** |  | **0.5%** | **0.8%** | **-** | |
| **Mean bias** | **-0.14** | **-0.59** | **-** | **-0.15** | **-0.14** | **-** | |
| **States closer to C-RC** | **16** | **11** | **-** | **17** | **10** | **-** | |

LE: Life expectancy

Equiv.: Equivalent deaths method

Conv.: Conventional method

Std. LT: Standard life table

RMSD: Root mean squared difference

**Figure A.1: Age-specific completeness of Brazil civil registry (%), equivalent deaths method (GBD std. LT), conventional method (GBD std. LT) and C-RC, by sex, Pará, Amapá and Maranhão, 2015 [2]**

**Figure A.1 (contd.)**

Equiv.: Equivalent deaths method

Conv.: Conventional method

Std. LT: Standard life table

**Table A.7: Age-specific completeness of Brazil civil registry (%), by state and sex, 2015, C-RC study [2]**

| **Age (years)** | **Acre** | | **Amazonas** | | **Roraima** | | **Pará** | | **Amapá** | | **Tocantins** | | **Maranhão** | |
| --- | --- | --- | --- | --- | --- | --- | --- | --- | --- | --- | --- | --- | --- | --- |
|  | **Male** | **Female** | **Male** | **Female** | **Male** | **Female** | **Male** | **Female** | **Male** | **Female** | **Male** | **Female** | **Male** | **Female** |
| 0 | 86 | 90 | 74 | 73 | 71 | 68 | 56 | 56 | 68 | 67 | 82 | 83 | 47 | 52 |
| 1-4 | 79 | 92 | 76 | 77 | 88 | 70 | 64 | 69 | 85 | 70 | 88 | 88 | 57 | 46 |
| 5-9 | 100 | 87 | 87 | 80 | 79 | 79 | 75 | 81 | 75 | 88 | 94 | 80 | 55 | 64 |
| 10-14 | 100 | 100 | 85 | 77 | 76 | 93 | 77 | 79 | 80 | 89 | 94 | 85 | 64 | 63 |
| 15-19 | 88 | 88 | 92 | 95 | 87 | 95 | 78 | 84 | 83 | 73 | 87 | 92 | 76 | 68 |
| 20-24 | 89 | 100 | 90 | 96 | 86 | 82 | 82 | 84 | 82 | 86 | 92 | 87 | 77 | 76 |
| 25-29 | 94 | 93 | 92 | 92 | 90 | 83 | 84 | 88 | 79 | 95 | 92 | 95 | 78 | 83 |
| 30-34 | 98 | 94 | 91 | 93 | 93 | 89 | 85 | 88 | 84 | 80 | 88 | 92 | 80 | 84 |
| 35-39 | 94 | 97 | 89 | 94 | 93 | 87 | 85 | 90 | 77 | 93 | 90 | 90 | 79 | 82 |
| 40-44 | 97 | 93 | 93 | 93 | 93 | 100 | 85 | 89 | 82 | 86 | 92 | 92 | 81 | 78 |
| 45-49 | 94 | 100 | 92 | 93 | 93 | 97 | 88 | 89 | 81 | 90 | 90 | 90 | 80 | 79 |
| 50-54 | 99 | 100 | 92 | 93 | 95 | 93 | 86 | 87 | 78 | 86 | 92 | 98 | 82 | 79 |
| 55-59 | 97 | 95 | 93 | 93 | 93 | 96 | 86 | 86 | 84 | 92 | 92 | 92 | 82 | 77 |
| 60-64 | 96 | 94 | 93 | 93 | 94 | 89 | 87 | 88 | 86 | 90 | 94 | 89 | 84 | 74 |
| 65-69 | 96 | 94 | 93 | 92 | 98 | 95 | 87 | 84 | 88 | 87 | 94 | 91 | 81 | 70 |
| 70-74 | 98 | 96 | 92 | 93 | 97 | 92 | 85 | 85 | 88 | 84 | 90 | 90 | 78 | 68 |
| 75-79 | 97 | 96 | 90 | 92 | 91 | 100 | 85 | 82 | 81 | 76 | 91 | 90 | 77 | 65 |
| 80-84 | 93 | 93 | 90 | 91 | 93 | 94 | 84 | 82 | 67 | 86 | 94 | 88 | 72 | 64 |
| 85+ | 92 | 95 | 83 | 88 | 92 | 97 | 75 | 82 | 76 | 79 | 87 | 86 | 69 | 61 |
| **All** | **94** | **95** | **89** | **90** | **91** | **91** | **82** | **82** | **80** | **83** | **91** | **89** | **76** | **68** |

**Table A.7 (contd.)**

|  | **Piauí** | | **Ceará** | | **Rio Grande do Norte** | | **Bahia** | |
| --- | --- | --- | --- | --- | --- | --- | --- | --- |
| **Age (years)** | **Male** | **Female** | **Male** | **Female** | **Male** | **Female** | **Male** | **Female** |
| 0 | 50 | 47 | 79 | 80 | 76 | 75 | 74 | 77 |
| 1-4 | 86 | 74 | 89 | 83 | 77 | 93 | 83 | 81 |
| 5-9 | 89 | 77 | 89 | 91 | 83 | 79 | 91 | 90 |
| 10-14 | 86 | 94 | 84 | 94 | 95 | 90 | 91 | 86 |
| 15-19 | 84 | 88 | 87 | 90 | 86 | 86 | 91 | 92 |
| 20-24 | 89 | 89 | 88 | 93 | 88 | 93 | 91 | 94 |
| 25-29 | 84 | 95 | 90 | 92 | 90 | 87 | 92 | 95 |
| 30-34 | 90 | 91 | 90 | 91 | 89 | 94 | 91 | 96 |
| 35-39 | 89 | 93 | 91 | 96 | 89 | 92 | 92 | 95 |
| 40-44 | 88 | 91 | 91 | 95 | 88 | 94 | 91 | 94 |
| 45-49 | 88 | 93 | 92 | 95 | 89 | 91 | 93 | 94 |
| 50-54 | 92 | 92 | 93 | 95 | 89 | 92 | 94 | 94 |
| 55-59 | 92 | 89 | 94 | 95 | 90 | 92 | 94 | 94 |
| 60-64 | 94 | 91 | 95 | 96 | 93 | 88 | 94 | 94 |
| 65-69 | 93 | 90 | 95 | 95 | 92 | 91 | 95 | 92 |
| 70-74 | 92 | 89 | 95 | 93 | 91 | 89 | 94 | 92 |
| 75-79 | 89 | 88 | 94 | 93 | 92 | 86 | 94 | 92 |
| 80-84 | 92 | 86 | 94 | 92 | 90 | 85 | 94 | 92 |
| 85+ | 89 | 85 | 92 | 92 | 90 | 86 | 90 | 91 |
| **All** | **89** | **86** | **92** | **93** | **90** | **87** | **92** | **92** |

Std. LT: Standard life table

**Table A.8: Male *_45_q_15_* according to equivalent deaths method (GBD and UN std. LT), conventional method (GBD std. LT), C-RC, Queiroz, IBGE and Bayesian [3]**

|  | **Equiv. (GBD std. LT) 2015** | **Conv. (GBD std. LT) 2015** | **Equiv. (UN std. LT) 2015** | **C-RC 2015** | **Queiroz 2000-10** | **IBGE 2000-10** | **Bayesian 2010** |
| --- | --- | --- | --- | --- | --- | --- | --- |
| Acre | 0.198 | 0.197 | 0.190 | 0.185 | 0.229 | 0.240 | 0.209 |
| Amazonas | 0.195 | 0.187 | 0.189 | 0.188 | 0.196 | 0.248 | 0.212 |
| Roraima | 0.202 | 0.201 | 0.196 | 0.197 | 0.241 | 0.271 | 0.213 |
| Pará | 0.186 | 0.184 | 0.179 | 0.191 | 0.227 | 0.249 | 0.240 |
| Amapá | 0.184 | 0.186 | 0.178 | 0.197 | 0.218 | 0.209 | 0.208 |
| Tocantins | 0.174 | 0.183 | 0.166 | 0.181 | 0.199 | 0.234 | 0.220 |
| Maranhão | 0.186 | 0.208 | 0.178 | 0.200 | 0.193 | 0.293 | 0.221 |
| Piauí | 0.200 | 0.200 | 0.192 | 0.194 | 0.183 | 0.272 | 0.200 |
| Ceará | 0.203 | 0.208 | 0.194 | 0.207 | 0.207 | 0.242 | 0.216 |
| Rio Grande do Norte | 0.180 | 0.190 | 0.171 | 0.199 | 0.183 | 0.207 | 0.209 |
| Bahia | 0.197 | 0.211 | 0.188 | 0.208 | 0.212 | 0.251 | 0.236 |
| Rondônia | 0.195 | 0.179 | 0.189 | 0.177 | 0.228 | 0.243 | 0.225 |
| Paraíba | 0.200 | 0.205 | 0.190 | 0.210 | 0.217 | 0.262 | 0.235 |
| Pernambuco | 0.229 | 0.224 | 0.220 | 0.219 | 0.256 | 0.263 | 0.251 |
| Alagoas | 0.228 | 0.232 | 0.220 | 0.235 | 0.245 | 0.296 | 0.284 |
| Sergipe | 0.229 | 0.230 | 0.221 | 0.234 | 0.226 | 0.258 | 0.235 |
| Minas Gerais | 0.179 | 0.186 | 0.170 | 0.182 | 0.213 | 0.190 | 0.213 |
| Espírito Santo | 0.181 | 0.191 | 0.173 | 0.189 | 0.233 | 0.200 | 0.231 |
| Rio de Janeiro | 0.213 | 0.201 | 0.205 | 0.199 | 0.259 | 0.211 | 0.239 |
| São Paulo | 0.189 | 0.175 | 0.181 | 0.171 | 0.224 | 0.178 | 0.199 |
| Paraná | 0.191 | 0.186 | 0.184 | 0.183 | 0.214 | 0.190 | 0.218 |
| Santa Catarina | 0.177 | 0.162 | 0.170 | 0.155 | 0.197 | 0.158 | 0.185 |
| Rio Grande do Sul | 0.191 | 0.181 | 0.183 | 0.178 | 0.208 | 0.177 | 0.201 |
| Mato Grosso do Sul | 0.188 | 0.187 | 0.181 | 0.188 | 0.220 | 0.205 | 0.215 |
| Mato Grosso | 0.189 | 0.193 | 0.183 | 0.198 | 0.229 | 0.218 | 0.226 |
| Goiás | 0.205 | 0.204 | 0.197 | 0.203 | 0.226 | 0.211 | 0.226 |
| Distriro Federal | 0.167 | 0.155 | 0.162 | 0.149 | 0.191 | 0.171 | 0.181 |
| **Correlation coefficient with:** |  |  |  |  |  |  |  |
| Queiroz 2000-10 | 0.619** | 0.442* | 0.629** | 0.423* | - | 0.188 | 0.649** |
| IBGE 2000-10 | 0.589** | 0.759** | 0.585** | 0.720** | 0.188 | - | 0.619** |
| Bayesian 2010 | 0.689** | 0.771** | 0.677** | 0.800** | 0.649** | 0.619** | - |
| **Average** | **0.632** | **0.657** | **0.630** | **0.648** | **0.418** | **0.403** | **0.634** |

*p<0.05 **p<0.01

Equiv.: Equivalent deaths method

Conv.: Conventional method

Std. LT: Standard life table

**References**

1. GBD Mortality Collaborators: **Global, regional, and national under-5 mortality, adult mortality, age-specific mortality, and life expectancy, 1970-2016: a systematic analysis for the Global Burden of Disease Study 2016.** *Lancet* 2017, **390:**1084-1150.

2. Costa LFL, Montenegro MdMS, Neto DdLR, de Oliveira ATR, Trindade JEdO, Adair T, Marinho MDM: **Estimating completeness of national and subnational death reporting in Brazil: Application of record linkage methods.** *Population Health Metrics* 2020, **18**.

3. Queiroz BL, Gonzaga MR, Vasconcelos AMN, Lopes BT, Abreu DMX: **Comparative analysis of completeness of death registration, adult mortality and life expectancy at birth in Brazil at the subnational level.** *Popul Health Metr* 2020, **18:**11.
